# Status of Ebola Virus Disease (EVD) preventive practices among Health care workers (HCWs) in Benin City: a year after disease containment in Nigeria

**DOI:** 10.11604/pamj.2018.30.50.15594

**Published:** 2018-05-21

**Authors:** Amenze Oritsemofe Onowhakpor, Vincent Yakubu Adam, Oghenetega Ewomazino Sakpa, Lydia Ukamaka Ozokwelu

**Affiliations:** 1 Department of Community Health, College of Medical Sciences, School of Medicine, University of Benin, Benin City, Nigeria

**Keywords:** Ebola virus disease, health care workers, knowledge, attitude, preventive practices

## Abstract

**Introduction:**

Ebola Virus Disease (EVD) is of great public health importance. Health care workers (HCWs) in various health care facilities especially in developing countries such as Nigeria, are particularly vulnerable to the disease. It is therefore imperative that health care workers adopt the necessary preventive practices to reduce to the barest minimum the risk of infection transmission. The study assessed the factors associated with EVD preventive practices among HCWs in a tertiary institution in Benin City, Nigeria.

**Methods:**

A total of 374 health care workers selected using a two-staged sampling technique participated in this descriptive cross-sectional study. Data were collected using structured, self-administered questionnaires and analyzed with IBM SPSS, version 21.0. Univariate and bivariate analysis were done. Level of significance was set at p < 0.05.

**Results:**

The mean age of respondents was 36.3 ± 8.0 years. All, 374 (100.0%) of the respondents were aware of EVD and 347 (92.8%) of respondents had good knowledge of EVD. More than half of the respondents, 228 (61.0%) and 201 (53.7%) had positive attitude towards EVD and good EVD preventive practices respectively. A higher proportion of respondents with positive attitude towards EVD were observed to have good preventive practice (p < 0.05).

**Conclusion:**

Knowledge, attitude and preventive practices towards EVD among HCWs were generally good. Positive attitude towards EVD was significantly associated with good preventive practices.

## Introduction

Ebola Virus Disease (EVD) is an acute viral disease resulting from infection with one of the Ebola virus strains a member of the filo virus family [1,2]. It is highly infectious with a high case fatality rate of 50-90% depending on the subtype [3,4]. Transmission occurs through infected body fluids and secretions of both living and dead people [3]. Early non-specific symptoms which may resolve in some survivors or may progress to full blown haemorrhagic syndrome are characteristic features of the disease [5-7]. There is presently no cure or vaccine for the disease although there are ongoing evaluations of drug therapies [8]. The largest ever outbreak of EVD was recorded in West Africa between 2014 and 2015. The outbreak spread from 4 West African countries (Guinea, Liberia, Sierra Leone and Nigeria) to Europe and America causing huge socioeconomic impact [2,9]. The outbreak in Nigeria began on the 20^th^ of July 2014 and spanned through to 19^th^ October 2014, the date Nigeria was declared Ebola free. In Nigeria, the outbreak recorded 11 confirmed cases and 5 deaths among health care workers [10]. As at 29^th^ of March 2016, 28,616 confirmed, probable and suspected cases were reported in Guinea, Liberia and Sierra Leone with 11,310 deaths. The outbreak declared over on the 9^th^ June 2016 [11]. HCWs are susceptible to this disease because of the nosocomial spread that has been identified as a major mode of transmission from human to human. This is enhanced by poor knowledge, poorly equipped health facilities to diagnose patient at the early stages of the disease [12-16] and non-compliance with standard precautions due to lack of personal protective equipment (PPEs) in health facilities. Knowledge is an easy cost effective method of EVD prevention in the advent of no current cure [8]. Thus the need to study the current knowledge, attitude and practice alongside the factors associated with preventive practices of EVD among HCWs in a tertiary health care institution. This will help develop interventions targeted at the gaps identified. In addition, this study is timely as it was conducted about a year after the end of the outbreak in Nigeria, to understand the changes that may have occurred in terms of knowledge, attitude and preventive practices among HCWs who are the frontline responders to medical emergencies.

## Methods

A cross-sectional descriptive study was conducted from July 2015 to October 2015 at the University of Benin Teaching Hospital (UBTH), Edo State, Nigeria approximately a year after Nigeria was declared EVD free. UBTH is a tertiary health facility located in the Egor Local Government Area of Edo State, providing primary, secondary and tertiary care to its environs and training of high and middle level manpower for the health industry. The hospital has thirty-three departments and offers a wide range of services. As at the time of the study, the number of doctors, nurses and laboratory scientists employed by UBTH were 758,816, and 163 respectively. The study was carried out among consenting HCWs (medical doctors, nurses and laboratory scientist) who had been employees at the hospital for more than 6 months The sample size for this study was determined using Cochran's formula for simple proportion [17]. In this study, p was taken as 67% which was the proportion of HCWs who were knowledgeable about EVD in a Lagos based study. Allowing for a non-response rate of 10%, the minimum sample size was 374 [18]. A two-staged sampling technique was used for selection of respondents. Stage 1-There are a total number of 33 departments in the hospital, out of which 23 are clinical departments comprising of the required study participants (doctors, nurses and laboratory scientist). Twelve departments were selected from the 23 using a simple random sampling technique by balloting. Stage 2-Departments were selected using a stratified random sampling technique. HCWs in each professional group made up a stratum. The number of HCWs in each profession stratum was obtained from the hospital management (Medical doctors: 758; Nurses: 816; Laboratory scientist: 163) The number of respondents utilized for the study in each stratum was subsequently calculated using the formula, sampling fraction × number of HCWs in a stratum. Where, sampling fraction = sampling size/total population. The total number of HCWs utilised for the study were as follows: medical doctors = 163; nurses = 176 and laboratory scientist = 35. Proportional allocation to size was then used to calculate the number of health workers per stratum in each department. Sampling framed consisted of a seperate list of doctors, nurses and laboratory scientist in each selected department. A systematic sampling technique was used to select respondents in each stratum. Data required for this study was collected with the use of structured self-administered questionnaire. The questions consisted of the following sections: socio-demographic profile of respondents, knowledge of EVD of respondents, attitude of respondents towards EVD, preventive practices of respondents towards EVD.

Data were analyzed using an electronic statistical package IBM SPSS version 21.0. Univariate and bivariate analysis were done. Quantitative data like, socio-demographic characteristics of respondents were presented as frequency tables, while continuous variables that were normal in distribution (such as age) were expressed as mean (standard deviation). T-test was used to compare the mean ages of male and female. Chi-square statistical test of association was used to determine the association between independent variables (socio-demographic characteristic, knowledge of EVD and attitude towards EVD among respondents) and dependent variable (preventive practices among respondents). Fisher's exact test was also used to compare associations when more than 20.0% of the expected cells had values less than 5. Level of significance was set at p < 0.05. Knowledge of EVD was assessed using a total of 50 questions addressing the following domains- awareness of EVD, causative agent, mode of transmission, symptoms, laboratory findings, prevention and treatment A score of “1” was given for correct response, and “0” for incorrect response. The total knowledege score obtained was converted to percentage. A score equal to or greater than 70.0% was classified as good knowledge, while a score of 50.0 to 69.9% was classified as fair knowledge and a score less than 50.0% was classified as poor knowledge. Attitude towards EVD was assessed using a total of 28 questions. Questions focused on attitude towards suspected, probable or confirmed case, risk perception, prevention and control practices. A score of “1” was given for correct response and “0” for incorrect response. The total attitude score obtained was converted to percentage. The maximum achievable score was 100% and the minimum 0%. A score equal to or greater than 70.0% was classified as positive attitude, while a score of 0 to 69.9% was classified as negative attitude. Preventive practices towards EVD were assessed using a total of 13 questions. The questions focused on hand washing, use of PPEs, hospital waste management, monitoring and surveillance, training and re-training of HCWs. A score of “1” was given for correct response and “0” for incorrect response. The total practice score obtained was converted to percentage. Percentage scores were graded as 0 to 49.9% as poor, 50.0% to 69.9% as fair and ≥ 70.0% as good practice. Ethical approval to conduct the study was obtained from the University of Benin Ethical and Research Committee. Informed written consent was obtained from participants.

## Results

One hundred and eighty six (49.7%) of the respondents were within the age group 31-40 years with a mean age was 36.4 ± (8.0) years. Two hundred and forty three (65.0%) of the respondents were females. Two hundred and three (54.3%) of respondents were married while 5 (1.3%) were co-habiting. One hundred and seventy six (47.1%) of the respondents were nurses, while 35 (9.4%) were laboratory scientists. One hundred and sixty (42.8%) respondents had less than five years duration of practice while 74 (19.8%) had practiced for >10 years. Mean duration of practice was 7.52 ± (6.4) years (Table 1). All the respondents were aware of EVD. The media was the source of information on EVD for 340 (90.0%) of the respondents followed by doctors 280 (74.9%). Others HCW, friends and family were mentioned by 273 (73.0%), 227 (60.7%) and 215 (57.5%) respectively of the respondents. Three hundred and sixty four (97.3%) respondents identified the causative agent of EVD as a virus. Concerning symptoms of EVD, 367 (98.1%) respondents reported fever as a symptom. Three hundred and seventy (98.1%) respondents said EVD can be prevented. Majority 303 (81.0%) of respondents said they had heard about notification of febrile illnesses. Majority 347 (92.8%) had an overall good knowledge of EVD (Table 2, Table 2 (suite)). Two hundred and twenty respondents (61.0%) of the respondents had a positive attitude towards EVD with 146 (39.0%) respondents had a negative attitude. Three hundred and twenty one (85.8%) of the respondents said they had received training on infection control while 279 (74.6%) of them had received training on EVD. Two hundred and sixty (69.6%) of the respondents opined that preventive facilities was provided by the hospital. Majority 343 (91.7%) of respondents said they always disposed waste safely in appropriate containers always. Three hundred and eleven (83.2%) of the respondents opined that they always washed their hands with soap and water (Table 3, Table 3 (suite)). A higher proportion 145 (63.6%) of the respondents with positive attitude towards EVD, also had good EVD preventive practices, while less than half 58 (39.7%) of respondents with negative attitude also had poor EVD preventive practices. This was statistically significant (p < 0.001) (Table 4). One hundred and seventy four (54.9%) of the respondents said they faced several challenges in adhering to proper preventive practices. Majority, 122 (70.1%) of the respondents said inadequate PPE's was a constraints. Other constraints mentioned were lack of training 24 (13.8%), bureaucratic bottle necks 18 (10.4%) and lack of an isolation unit 10 (5.7%).

**Table 1:** Socio-demographic characteristics of respondents

Socio-demographic characteristics	Frequency n= 374	Percent
**Age (years)**		
21-30	88	23.5
31-40	186	49.7
41-50	72	19.3
51-60	28	7.5
**Mean age= 36.3(±8.0) years**		
**Mean age (female) = 37.4 ± (8.0) years**		
**Mean age (male) = 34.4 ± (7.7) years**		
**t test = 3.576 p=0.83**		
**Sex**		
Female	243	65.0
Male	131	35.0
**Marital status**		
Single	142	38.1
Married	203	54.3
Separated	11	2.9
Divorced	5	1.3
Widowed	8	2.1
Cohabiting	5	1.3
**Profession**		
Nurse	176	47.1
Medical doctor	163	43.6
Laboratory scientist	35	9.4
**Duration of practice (years)**		65.0
<5	160	35.0
5 – 10	140	
>10	74	38.1

Mean duration of practice = 7.5± 6.4 years

**Table 2:** Respondents’ knowledge of EVD

Knowledge	Freq (n = 374)	Percent
Mode oftransmission*		
Contact withsweat	363	97.1
Direct physical handling of corpse	363	97.1
Contact withblood	362	96.8
Handling and eating bush meat	359	96.0
Contact withurine	357	95.5
Contact withsemen	356	95.2
Contact withsaliva	355	94.9
Transfusion of infected blood and blood products	355	94.9
Contact withbat	354	94.7
Sharing sharps with infected person	350	93.6
Contact withbreast milk Contact withmonkey Contact withgorilla	345 305 250	92.2 81.6 66.8
Contact with forest antelope	225	60.2
Contact with porcupine	70	18.7
Contact with rats	43	11.5
Mosquito bite	39	10.4
Air	17	4.5
**Causative agent of EVD**		
Virus	364	97.3
Protozoa	6	1.6
Bacteria	4	1.1
Symptoms*		
Fever	367	98.1
Vomiting	361	96.5
Diarrhoea	358	95.7
Bleeding	356	95.2
Joint pain	339	90.6
Muscle pain	339	90.6
Fatigue	335	89.6
Rash	332	88.8
Sore throat	331	88.5
Stomach pain	330	88.2
Headache	325	86.9
Red eyes	300	80.2
Laboratory findings*		
Low platelet count	307	82.1
Elevated liver enzymes	267	71.4
Low WBC	261	69.8

**Table 2 (suite):** Respondents’ knowledge of EVD

Knowledge	Freq (n = 374)	Percent
**EVD canbeprevented**		
Yes	370	98.9
No	4	1.1
**Prevention of EVD ( n=370)^+^**		
Proper handling of corpse using protective wear	359	97.0
Isolation of suspected cases	357	96.5
Seeking prompt medical attention for suspected cases	357	96.5
Avoiding direct contact with body fluid	353	95.4
Disinfecting items of Ebola patients	350	94.6
Prompt and safe burial of the dead	346	93.5
Prompt reporting of suspected cases	343	92.7
Regular hand washing	343	92.7
Bathing with salt and water	9	2.4
**Prevention in health care setting^+^**		
Use of PPE’s	359	96.0
Regular hand washing	356	95.2
Properhandling of suspected cases by trained personnel	355	94.9
Safe burial practices Safe injection practices	350 338	93.6 90.4
Vaccine	176	47.1
**Treatment^+^**		
IV fluid	311	83.2
Isolation	291	77.8
Use of drugs	188	50.3
Traditional and spiritual healers	13	3.5
**Ebola can be cured**		
Yes	183	49.9
No	191	50.1
**Heard about notification of febrile illnesses**		
Yes	303	81.0
No	71	19.0
**Overall knowledge**		
Good	347	92.8
Fair	17	4.5
Poor	10	2.7

**Table 3:** EVD preventive practices among respondents

Preventive practices	n (%)	
	Yes	No
Received training on infection control	321 (85.5)	53 (14.2)
Report all suspected cases of EVD	310 (82.9)	64 (17.1)
Isolate all suspected cases of EVD	308 (82.4)	66 (17.6)
Received training on EVD	279 (74.6)	95 (25.4)
Use complete PPE for EVD management	210 (56.1)	164 (43.9)
Take precaution when handling diseased patients	210 (56.1)	164 (43.9)
Face constraints adhering to EVD preventive practices	174 (46.5)	200 (53.5)
**Provision of preventive services**		
Hospital	260	69.6
Self	60	16.0
NGOs	45	12.0
Colleagues	9	2.4
**Channel of communication on discovery of a suspected case**		
Infection control unit	174	46.5
Hospital management	124	33.2
Local government health team	76	20.3

**Table 3 (suite):** EVD preventive practices among respondents

Preventive practices	n (%)		
	Always	Sometimes	Never
Dispose waste safely in appropriate containers	343 (91.7)	20 (5.3)	11 (2.9)
Wash hands with soap and water	311 (83.2)	51 (13.6)	12 (3.2)
Wash hand before and after eating	300 (80.2)	59 (15.8)	15 (4.0)
Use hand sanitizers	207 (55.3)	144 (38.5)	23 (6.2)
Wear gloves and PPEs	192 (51.3)	167 (44.7)	15 (4.0)

**Table 4:** Determinants of EVD preventive practices among respondents

	Preventive practices			Test statistic
Variable	Good n (%)	Fair n (%)	Poor n (%)	
**Age (years)**				
≤ 40	152 (55.5)	47 (17.2)	75 (27.4)	χ^2^ = 1.243
> 40	49 (49.0)	20 (20.0)	31 (31.0)	p = 0.537
**Sex**				
Male	60 (45.8)	26 (19.8)	45 (34.4)	χ^2^ = 5.355
Female	141 (58.0)	41 (16.9)	61 (25.1)	p = 0.069
**Marital status**				
Ever married	130 (57.3)	40 (17.6)	57 (25.1)	χ^2^ = 5.421
Never married	71 (48.3)	27 (18.4)	49 (33.3)	p = 0.174
**Profession**				
Nurse	100 (56.8)	33 (18.8)	43 (24.4)	χ^2^ = 3.892
Doctor	86 (52.8)	26 (16.0)	51 (31.3)	p = 0.421
Laboratory scientist	15 (42.9)	8 (22.9)	12 (34.3)	
**Duration of practice (years)**				
0 – 5	90 (56.3)	32 (20.0)	38 ( 23.8)	χ^2^ = 3.977
6 – 10	72 (51.4)	21 (15.0)	47 (33.6)	p = 0.409
>10	39 (52.7)	14 (18.9)	21 (28.4)	
**Knowledge**				
Good	189 (54.5)	63 (18.2)	95 (27.4)	Fishers Exact = 7.496
Fair	10 (58.8)	3 (17.7)	4 (23.5)	p = 0.062
Poor	2 (20.0)	1 (10.0)	7 (70.0)	
**Attitude**				
Positive	145 (63.6)	35 (15.4)	48 (21.1)	χ^2^ = 23.644
Negative	56 (38.4)	32 (21.9)	58 (39.7)	p < 0.001

## Discussion

All the respondents were aware of the EVD as was anticipated due to the 2014 EVD epidemic and sensitization of all Nigerians on the deadly disease following the outbreak. This high level of awareness among HCWs may also be attributed to the education and training on EVD and its preventive measures owing to the nature of their profession. This is in line with findings from a study done in Lagos and Ile- Ife, Nigeria and Ethiopia where 85.5%, 95% and 94.6% respectively of the HCWs were aware of EVD [14,18,19]. The major source of information on EVD was the media. The social media played a huge role in the Ebola epidemic as it helped to improve early warning systems, outbreak response and communication between healthcare providers. This is in tandem with findings from a study done in Ethiopia [19]. This is of public health significance as more recently, the rapid global reach in media and internet access has created a means for public health professionals to communicate effectively and to gain insight into emerging disease events. Overall, majority of the respondents had a good knowledge of EVD. This was in accordance with findings observed in two studies conducted in Lagos among HCWs which revealed that 72.5% and 98.5% respondents respectively had good knowledge of EVD [18,20]. Contrasting findings were seen in similar studies done in Ile-Ife and Maiduguri which reported low levels of good knowledge (42.3% and 40.9% respectively) among respondents [14,21]. About two-thirds of the respondents had a positive attitude towards EVD. This was similar to findings observed in a study done in Lagos, Nigeria which reported positive attitude among 67% of the respondents [20]. Contrasting findings were seen in Shimoga, Central Karnataka with 12.8% of the respondents having very good attitude towards EVD [19]. Nonetheless, good knowledge is vital in improving the attitude of HCWs towards EVD. Majority of respondents had received training on EVD and infection control. Despite this, available PPEs were still not optimally used. This may be due to the fact that most respondents had never managed or handled an EVD patient.

This is problematic as non-use of PPEs increases the risk of acquisition of nosocomial infections among HCWs which can further spread to their families and the larger society. In addition, this study revealed a deficiency of PPE kits as only half of respondents agreed that complete PPE kits were made available for their use. This finding is of public health importance as it further emphasises the need to establish the routine practice of standard precautions among HCWs as opportunities for transmission of EVD to personnel in health facilities exist mainly because standard precautions are usually not followed. Overall, majority of the respondents had good practice towards EVD prevention. Good practice was also observed among 93.8% of HCWs in public facilities, in a study in Lagos, Nigeria [20]. This was consistent with a similar study carried out among health care professionals in Shimoga, Central Kernataka where only 8% of the respondents had very good practice as regards personal protection, safety issues and biomedical waste management [22]. Good knowledge and attitude towards EVD could have translated into good practice. Attitude was found to be the only statistically significant factor associated with adherence to preventive practices towards EVD among respondents. This is implies that a positive attitude towards EVD is of vital importance in ensuring good preventive practices among HCWs. Attitude of HCWs towards suspected, probable or confirmed cases alongside their risk perception influences proper adherence to preventive practices. This is of great significance in the prevention and control of spread of EVD. Majority of respondents reported inadequate and incomplete PPEs as a constraint to adhering to EVD preventive practices. Other constraints to good preventive practices reported in this study included; lack of training on their use, lack of time and indifference. This was found to be so, because there are no stringent measures to ensure compliance with the use of available protective measures and facilities. This was similar to the study in Central Kernataka where factors influencing good preventive practices included; lack of knowledge (24%), and lack of motivation (21.5%), lack of supplies (19.5%) and lack of time (15%) [22], another study highlighted busy schedules, non-use by colleagues and discomfort as barriers to use [23].

**Study limitations**: One of the limitations is the cross-sectional study design. In addition generalization of the study findings to other setting in the study locale is limited because only one tertiary health institution was utilized.

## Conclusion

Knowledge, attitude and preventive practices towards EVD among HCWs were generally good. Positive attitude towards EVD was significantly associated with good preventive practices. The main challenge reported by respondents in adhering to preventive practices was inadequate and incomplete PPEs. There is need for management to focus on provision of personal protective equipment's as well as training and re-training of staff to improve attitude of staffs so that standard precautions are adhered to. This will consequently prevent the spread of infectious diseases in the healthcare setting.

**Recommendations**: Gaps in attitude and preventive practices identified provides rational for training and re-training of staff to improve attitude of staffs so that standard precautions can be practiced optimally in the health setting. Personal protective equipment's should also be made readily available and accessible to health care workers to enhance use.

### What is known about this topic

- Previous studies dwelt on the knowledge, attitude and preventive practices of health care workers towards Ebola virus disease and these researches mainly took place during or after the peak period of the outbreak;
- They revealed varying knowledge and attitude towards Ebola virus disease in different settings and sub-populations.

### What this study adds

- This research was conducted about a year after Nigeria was certified Ebola virus disease free to determine if the situation have changed overtime in terms of health care workers knowledge, attitude and preventive practices towards Ebola virus disease;
- The study revealed that knowledge level still remains high: sixty-one percent of the health care workers had positive attitude towards Ebola virus disease;
- However, adherence to preventive practices is suboptimal and shortage of personal protective equipment does still exist as major challenges.
